# *BIM*基因多态性对晚期肺腺癌一线EGFR-TKIs疗效影响回顾性研究

**DOI:** 10.3779/j.issn.1009-3419.2017.08.07

**Published:** 2017-08-20

**Authors:** 坤 钱, 毅 张, 修益 支

**Affiliations:** 100053 北京，首都医科大学宣武医院胸外科，首都医科大学肺癌诊疗中心 Department of Thoracic Surgery, Xuanwu Hospital, Diagnostic and Treatment Centers of Lung Cancer, Capital Medical University, Beijing 100053, China

**Keywords:** 肺肿瘤, 酪氨酸激酶抑制剂, 多态性, *BIM*基因, Lung neoplsms, Tyrosine kinase inhibitor, Polymorphism, *BIM* gene

## Abstract

**背景与目的:**

通过对85例肺腺癌患者石蜡包埋标本及部分全血样本*BIM*缺失多态性的检测，分析*BIM*多态性与酪氨酸激酶抑制剂（tyrosine kinase inhibitors, TKIs）药物疗效相关性，初探不同类型标本BIM检测的相关性。

**方法:**

收集2013年2月-2014年11月间经宣武医院胸外科诊断明确的Ⅲb期-Ⅳ期肺腺癌患者，表皮生长因子受体（epidermal growth factor receptor, EGFR）19或21外显子突变85例，给予一线TKIs治疗，采用石蜡组织标本和部分全血进行*BIM*基因多态性检测，分析两组患者治疗客观有效率（objective response rate, ORR）、无进展生存期（progression-free survival, PFS），并根据吸烟、性别、*EGFR*突变位点等因素进行单因素分析，同时对比石蜡标本与血液检测BIM的相关性。

**结果:**

在受检的85例FFPE样本中，*BIM*基因具有缺失多态性14例（16.47%），纯和无缺失多态性71例（83.53%）。在13例对照样本中，石蜡样本和血液样本检出*BIM*基因缺失多态性2例，且为相同患者样本。BIM多态性的患者在用药物后的客观缓解率与无多态性组无统计学差异（*P*>0.05）。*BIM*基因缺失多态性、纯和无缺失患者接受药物治疗的中位PFS分别为7.1个月、12.8个月，存在统计学差异（*P*=0.013）。男性和女性中位PFS（10.7个月、12.1个月，*P*=0.835）、吸烟组和非吸烟组中位PFS（9.7个月、12.1个月，*P*=0.974）、EGFR 19和21外显子中位PFS（8.7个月、12.2个月，*P*=0.303）比较均无统计学差异（*P*>0.05）。

**结论:**

检测患者BIM基因多态性对晚期肺腺癌EGFR-TKIs治疗患者的评估预后可能有一定参考意义，但需要进行大样本的研究。

## Abstract (en)

**Background and objective:**

The aim of this study is to detect the *BIM* polymorphism in 85 formalinfixed and parrffin-embedded (FFPE) and some blood samples of advanced lung adenocarcinoma patients and study the relativity betweenthe BIM polymorphism and tyrosine kinase inhibitor (TKI). The correlation between BIM detection of different types of specimens was discussed.

**Methods:**

There were 85 patients who were diagnosed as advanced lung adenocarcinoma with epidermal growth factor receptor (*EGFR*) 19 or 21 exon mutation in thoracic surgery of Xuanwu Hospital from February 2013 to November 2014, all of who were received EGFR-TKI as first-line treatment in the study. FFPE and some blood were used to detect the BIM polymorphism. The objective response rate (ORR) and progression-free survival (PFS) of two groups were compared. According to smoking, sex, *EGFR* mutation and other factors, the single factor analysis was performed, and the correlation between paraffin samples and blood test BIM was compared.

**Results:**

The ORR in *BIM* polymorphism and nonpolymorphism groups was no significant differences (*P*>0.05). The median PFS in *BIM* polymorphism and non-polymorphism group was 7.1 months and 12.8 months, respectively (*P*=0.013). Univariate analysis the median PFS, women were longer than men (12.1 months *vs* 10.7 months, *P*=0.835); Non-smokers were longer than smokers (12.1 months *vs* 9.7 months, *P*=0.974). Group in EGFR exon 21 is longer than group in EGFR exon 19 (12.2 months *vs* 8.7 months, *P*=0.303).

**Conclusion:**

Detection of *BIM* gene polymorphism in lung cancer patients with EGFR-TKIs treatment might be helpful for predicting prognosis. But a large sample study is needed.

IPASS、OPITIMAL等试验奠定了表皮生长因子受体酪氨酸激酶抑制剂（epidermal growth factor receptor tyrosine kinase inhibitors, EGFR-TKIs）在有*EFGR*突变的晚期非小细胞肺癌患者中的一线治疗地位，在*EGFR*突变的人群中其有效率可达80%以上^[1, 2]^，但是仍然有20%左右的*EGFR*突变患者治疗效果并不理想，表现为原发性耐药或者无进展生存期（progression free survival, PFS）较短，许多研究^[3, 4]^提示可能存在其他信号通路和基因位点影响TKI的疗效。BCL-2蛋白家族是细胞凋亡进程中的重要调节蛋白，*BIM*基因作为BCL-2家族成员之一，是参与细胞凋亡的重要介质。研究^[5, 6]^发现东亚人群中*BIM*基因的2号内含子存在缺失多态性，导致患者会表达缺乏BH3结构域，即没有促凋亡活性的BIM亚型，从而引起该类患者对EGFR-TKIs的原发耐药或削弱TKIs的临床疗效。本研究通过对晚期肺腺癌患者的*BIM*多态性检测，分析*BIM*多态性与EGFR-TKIs药物疗效相关性，并探索不同类型标本BIM检测的相关性。

## 1 材料和方法

### 1.1 研究对象

本研究入选2013年2月-2014年11月间经宣武医院胸外科诊断明确的Ⅲb期-Ⅳ期肺腺癌患者85例，其中男性30例，女性55例；年龄范围35岁-82岁，中位年龄58岁；临床分期Ⅲb期34例，Ⅳ期51例；有吸烟史29例，无吸烟史56例（表 1）。所有患者均于接受TKI药物之前通过穿刺或者手术的方式取得病理组织。入选标准：①肿瘤-淋巴结-转移（tumor-node-metastasis, TNM）分期为Ⅲb期-Ⅳ期的肺腺癌，有EGFR基因19和/或21外显子敏感突变；②患者年龄18岁-80岁；③血液学、生化和器官功能：血红蛋白≥100 g/L；中性粒细胞绝对计数≥2.0×10^9^/L；血小板计数≥100×10^9^/L；总胆红素≤1.5倍正常值上限；谷丙转氨酶和谷草转氨酶≤2.5倍正常值上限；肌酐≤1.5倍正常值上限；且肌酐清除率≥60 mL/min；④美国东部肿瘤协作组（Eastern Cooperative Oncology Group, ECOG）行为状态评分0分-2分，预期寿命大于12周。排除标准：①既往合并恶性肿瘤病史；②已知对吉非替尼、厄洛替尼和埃克替尼中的任何成分过敏；③既往患间质性肺病，基线时计算机断层扫描（computed tomography, CT）扫描发现存在特发性肺纤维化；④任何不稳定的内科系统性疾病和感染性疾病；④已知的人类免疫缺陷病毒感染；⑤混有小细胞肺癌成份的患者；⑥怀孕或哺乳期妇女；

**1:** 患者的般临一床特征 General clinical characteristics of the patients

Item	Case	Proportion
Age (yr)		
> 58	42	49.41%
≤58	43	50.59%
Gender		
Male	30	35.29%
Female	55	64.71%
Stage		
Illb	34	40.00%
Ⅳ	51	60.00%
Smoking history		
Smoking	29	34.12%
No-smoking	56	65.88%

### 1.2 标本来源组织切片样本

所有样本均为首都医科大学宣武医院收集，病理明确诊断为肺腺癌，采用甲醛固定石蜡包埋（formalin-fixed and parrffin-embedded, FFPE）组织切片样本，5 μm厚切片不少于10片，室温存放。样本中的肿瘤组织至少占整个样本的30%。全血样本：使用EDTA抗凝剂的真空采血管，取不少于4 mL的全血，存放于2 oC-8 oC、24 h内送检。

### 1.3 治疗方法

患者根据不同情况治疗给予吉非替尼250 mg 1次/日或者厄洛替尼150 mg 1次/日或者埃克替尼125 mg 3次/日口服，因疾病进展或不能耐受的毒性反应而停止。患者服药后4周进行一次胸部CT平扫，根据实体瘤疗效评价标准（Response Evaluation Criteria in Solid Tumors, RECIST）标准进行判效，若肿瘤没有进展（progressive disease, PD）可继续服药，此后需每4周一次访视，口服药物8周后进行最终判效。服药期间每8周进行一次胸部CT扫描复查直至疾病进展或发生不能接受的毒性反应停药。

### 1.4 样本DNA提取

FFPE样本使用德国Qiagen公司的石蜡包埋组织DNA提取试剂盒提取FFPE样本基因组DNA，将病理组织从切片中提取，全血样本采用Qiagen公司血液基因组DNA提取试剂盒（离心柱型）提取全血样本基因组DNA，按照使用说明进行操作，石蜡样本和全血样本提取的DNA样品满足OD_260_/OD_280_=（1.8±0.2），OD_260_/OD_230_≥1.7；浓度为20 ng/μL-50 ng/μL。DNA于-20 oC贮存至使用。

### 1.5 *EGFR*基因突变检测

采用北京雅康博生物科技有限公司生产的人*EGFR*基因突变检测试剂盒（荧光PCR法），实时荧光PCR仪为Stratagene Mx3000P，循环设置为：95 oC、10 min；95 oC、15 s，60 oC、1 min，共40个循环。

### 1.6 *BIM*基因多态性检测

采用北京雅康博生物科技有限公司生产的人*BIM*基因多态性检测RUO试剂盒（荧光PCR法），实时荧光PCR仪为Stratagene Mx3000P，循环设置为：95 oC、10 min；95 oC、15 s，60 oC、1 min，共40个循环。

### 1.7 统计分析

通过SPSS 16.0进行统计分析，不同BIM结果的患者服药的客观有效率（objective response rate, ORR）间差异采用*Fisher’s*精确检验进行分析；多因素分析采用*Cox*回归；单因素分析采用*Kaplan-Meier*分析及*Log-rank*检验。生存曲线采用GraphPad Prism 5 Project软件制作。所有统计分析过程中，双侧检验*P* < 0.05为存在统计学差异。

## 2 结果

### 2.1 *EGFR*基因突变检测和*BIM*基因多态性结果

自2013年2月-2014年11月，收集EGFR 19、21外显子突变85例。其中19外显子突变35例，21外显子突变50例（图 1）。根据85例石蜡组织样本*BIM*基因多态性检测结果显示，*BIM*基因缺失14例（16.47%），纯和无缺失71例（83.53%）。在13例对照样本中，石蜡样本检出*BIM*基因缺失2例（15.4%）；血液样本检出*BIM*基因缺失2例（15.4%），且为相同患者样本。

**1:**
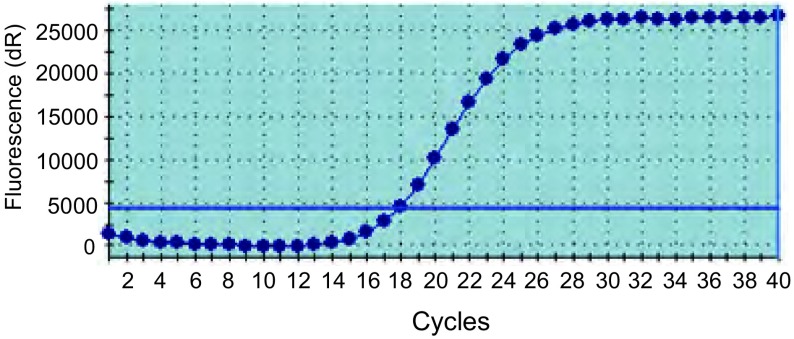
*EGFR*突变检测图谱 The amplification plots of *EGFR* mutation. EGFR: epidermal growth factor receptor

### 2.2 *BIM*基因多态性与一般临床特征的关系

*BIM*基因多态性与患者年龄、性别、分期及吸烟史均无统计学差异（*P* < 0.05）（表 2）。

**2:** *BIM*基因多态性与一般临床特征相关性分析 Relationship between *BIM* gene polymorphism and general clinical characteristics

Item	*BIM* deletion polymorphism	*BIM* non-deletion polymorphism	*P*
Age (yr)			0.255
> 58	9	33	
≤58	5	38	
Gender			0.762
Male	4	26	
Female	10	45	
Stage			0.388
Ⅲb	4	30	
Ⅳ	10	41	
Smoking history			0.363
Smoking	3	26	
No-smoking	11	45	

### 2.3 *BIM*基因多态性与短期疗效和无进展生存的关系

82例具备短期疗效数据的患者中，不同*BIM*多态性的患者在应用药物后的客观有效率无统计学差异（*P* < 0.05）（表 3）。在上述82例具备短期疗效数据的患者中，有67例患者的随访数据可供PFS分析。随后采用*Kaplan-Meier*曲线分析*BIM*基因多态性与该67例患者PFS之间的关系（图 2）。*BIM*基因缺失、无缺失患者接受药物治疗的中位PFS分别为7.1个月、12.8个月，且存在统计学差异（*P*=0.013）。

**3:** *BIM*多态性与短期疗效的差异 Relationship between *BIM* gene polymorphism and therapeutic efficacy of tyrosine kinase inhibitor

*BIM*	CR	PR	SD	PD	ORR (%)	*P*
Deletion polymorphism	0	4	8	2	28.57	0.557
Non-deletion polymorphism	1	26	36	5	39.71	
CR: complete response; PR: partial response; SD: stable dosease; PD: progressive disease; ORR: objective response rate.						

**2:**
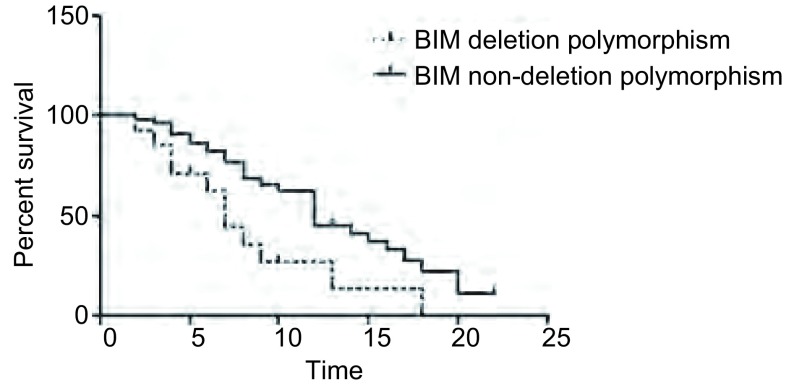
*BIM*基因多态性与无进展生存的关系 Single factor analysis of the relationship between PFS and *BIM* gene polymorphism. PFS: progression-free survival.

### 2.4 单因素分析PFS结果

从表 4可以看出*BIM*基因多态性、无多态性患者接受药物治疗的中位PFS分别为7.1个月、12.8个月，且存在统计学差异（*P*=0.013）。男性和女性中位PFS分别为10.7个月、12.1个月（*P*=0.835），无统计学差异（*P* < 0.05）；吸烟组和非吸烟组，中位PFS分别为9.7个月、12.1个月（*P*=0.974），无统计学差异（*P* < 0.05）；EGFR 19、21外显子中位PFS分别为8.7个月、12.2个月（*P*=0.303），无统计学差异（*P* < 0.05）。

**4:** 单因素与PFS相关性分析结果 Results of univariate analysis of PFS

Item	PFS (mo)	*X*^2^	*P*
*BIM*		6.201	0.013
*BIM* deletion polymorphism	7.1		
*BIM* non-deletion polymorphism	12.8		
Gender		0.043	0.835
Male	10.7		
Female	12.1		
Smoking history		0.001	0.974
Smoking	9.7		
No-smoking	12.1		
*EGFR* mutation		1.061	0.303
Exon 19	8.7		
Exon 21	12.2		

## 3 讨论

本研究85例FFPE样本*BIM*基因多态性检测结果显示，*BIM*基因缺失多态性14例（16.47%），Nakagawa等^[3]^研究报道*BIM*基因缺失多态性只存在于东方人群中，在*EGFR*基因突变的NSCLC患者中，*BIM*基因多态性率占12.9%。人类基因组单倍体图计划中的中国人*BIM*缺失多态性携带率为20.5%^[7]^。邓伟等^[5]^报道24.3%（42/169）的患者存在*BIM*缺失多态性。相比而言，德国等欧洲人群则未见*BIM*缺失多态性^[5]^。不同文献报道的BIM缺失多态性比例在12.3%-24.3%之间，本试验16.47%的阳性率符合文献报道范围之内。

*BIM*基因广泛分布于机体正常组织细胞，包括破骨细胞、成骨细胞、肥大细胞、上皮细胞、血管内皮细胞和神经细胞等正常组织中，在细胞凋亡中BIM是必不可少的，在恶性肿瘤细胞中亦有表达。在13例对照样本中，石蜡样本与血液样本检出2例结果相同的*BIM*基因缺失多态性，由于本研究使用的病理标本和全血标本均是患者服用EGFR-TKIs之前的标本，没有受到药物影响，初步证明血液样本检测BIM基因多态性的可行性，但是例数较少，且缺少患者用药后的对比检测，因此血液检测BIM多态性的可行性有待进一步验证。

从表 2也可以看出*BIM*基因多态性在患者年龄、性别、分期及吸烟史等因素中分布均无统计学差异（*P* < 0.05），也说明*BIM*多态性是广泛分布于人群中的一种随机性分布，不因患者性别等先天因素或者吸烟等后天环境所改变。但是有文献^[8]^报道某些药物如AZD6244能够增强FOXO_3_的表达，进一步提高BIM表达和诱导细胞凋亡。

从表 3可以看出82例具备短期疗效数据的患者中，*BIM*多态性组ORR为28.57%，而*BIM*无多态性组为39.71%，两组统计学上无显著差异。说明对于有*EGFR*突变的晚期肺腺癌，*BIM*多态性并不是一线使用TKIs治疗有效性的预测因素。郑蕾等^[9]^报道，在123例复治晚期非小细胞肺癌患者中，化疗失败后接受吉非替尼或厄洛替尼靶向治疗，其ORR和疾病控制率（disease control rate, DCR）在*BIM*多态性和无多态性人群中也无统计学意义。

本研究有67例患者的随访数据可供无进展生存分析，通过图 2可以看出*BIM*基因具有缺失多态性、无多态性患者接受药物治疗的中位PFS分别为7.1个月、12.8个月，且存在统计学差异（*P*=0.013），说明BIM多态性与TKI一线治疗晚期肺腺癌的长期疗效相关。由于BIM基因的2号内含子存在缺失多态性，导致产生没有促凋亡活性的BIM亚型，从而引起该类患者对EGFR-TKIs削弱的临床疗效，但是这一过程为何表现出只是PFS的差异而不是原发性耐药的方面的差异仍不得而知。Costa等^[10]^研究结果证实，对吉非替尼等TKIs有效的肺癌患者癌细胞内BIM表达数量显著增加，而BIM基因敲除后原来敏感的肿瘤细胞则对TKIs耐药，说明BIM表达水平在TKIs介导的肿瘤细胞凋亡过程中具有十分重要的作用。只有当BIM表达完全消失才会导致迅速耐药，而无论是*BIM*多态性还是低表达所引起的是一种缓慢性耐药，所引起的最终结果就是这类患者PFS缩短。至于*BIM*多态性与低表达之间的关系尚需要进一步试验以得出结论。

除了检验*BIM*基因的多态性与中位PFS的关系外，还将性别、吸烟状况和*EGFR*突变位点与PFS进行了独立的分析。虽然非吸烟组的PFS明显长于吸烟组，但是仍无统计学差异，性别和*EGFR*外显子敏感突变一样也不是PFS的独立预测因素，这一结果表明以前认为的“亚洲不吸烟女性腺癌”是EGFR-TKIs治疗优势人群可能是这类人群*EGFR*突变率较高，而并非是性别和吸烟这些因素决定的长期疗效^[1]^。

通过本回顾性研究可以得出以下结论：①*BIM*基因缺失多态性作为一种仅存在于东方人种中的突变随机分布，与性别等先天因素和吸烟等后天因素无关，不同文献报道的BIM缺失多态性比例在12.3%-24.3%^[5, 7, 9]^。②*BIM*基因多态性的有无对晚期肺腺癌一线EGFR-TKIs治疗患者的PFS有统计学差异，检测患者BIM基因多态性对晚期肺腺癌EGFR-TKIs治疗患者的评估预后有一定指导意义。但是*BIM*基因多态性的有无对晚期肺腺癌一线EGFR-TKIs治疗的短期疗效无法起到预测作用。③晚期肺腺癌一线EGFR-TKIs治疗患者的PFS与性别、吸烟状况等因素无关，*EGFR* 19和21突变位点在这类人群进行TKIs治疗中PFS也无统计学差异。④基于*BIM*多态性在体细胞和肿瘤细胞检测结果相同，虽然例数较少可以初探采用全血检测初治病人的*BIM*多态性结果，但复治患者的结果有待进一步验证。
